# When I say … team learning

**DOI:** 10.1111/medu.14174

**Published:** 2020-05-15

**Authors:** Stephanie N. E. Meeuwissen, Wim H. Gijselaers, Ineke H. A. P. Wolfhagen, Mirjam G. A. oude Egbrink

**Affiliations:** ^1^ School of Health Professions Education Faculty of Health, Medicine and Life Sciences Maastricht University Maastricht the Netherlands; ^2^ Department of Educational Development and Research Faculty of Health, Medicine and Life Sciences Maastricht University Maastricht the Netherlands; ^3^ Department of Educational Research and Development School of Business and Economics Maastricht University Maastricht the Netherlands; ^4^ Institute for Education Faculty of Health, Medicine and Life Sciences Maastricht University Maastricht the Netherlands; ^5^ Department of Physiology Faculty of Health, Medicine and Life Sciences Maastricht University Maastricht the Netherlands

## Abstract

Meeuwissen et al. present 'team learning' as a process that helps explain how successful teams work, clarifying how behavioral dimensions of team learning lead to new, shared understandings.

The way a team plays as a whole determines its success. You may have the greatest bunch of individual stars in the world, but if they don’t play together, the club won’t be worth a dime. Babe Ruth ^1^

The increasing complexity of health care systems creates a growing urgency to collaborate across disciplines. Insight into the underlying processes or, as described in the quote above ‘the way a team plays,’ is crucial to understand how teams synthesise and process profoundly different views of individuals. In successful teams, members think together and discuss each other's input, often resulting in an agreement, shared or new idea. Typically, in such teams, its members are highly engaged, feel safe to share innovative ideas and trust that asking critical questions is allowed. Moreover, they feel like they belong to a group, share responsibility amongst them and feel accountable for the group's outcomes. In case you ever experienced group meetings in such a way, you were likely part of a team demonstrating team learning behaviour. The need for such behaviour arises in educators’, researchers’ and physicians’ everyday practice, for example, when a complex patient case requires the input of different experts. Some patients may have heard their physician say something like:

I’ve seen the results of the scan. However, right now, I cannot give you an answer to what it means and what kind of treatment would be best. Later today, I'll discuss your case in our multidisciplinary team. Together, we'll discuss the severity of the disease and decide upon the best treatment that we can recommend to you considering your symptoms, test results and physical condition. I’ll come back to you so we can start treatment accordingly.

Sadly though, not every group is able to reach this state of team learning, which may affect the quality of health care in a detrimental way. Navigating the organisational behaviour literature, we find that Edmondson^2^ already pointed out that a team's success or failure depends on how its team members interact and create a working climate that makes it possible for the team to ‘learn’. She introduced the concept of ‘team learning’ as a collective discourse activity that teams undertake to obtain new insights and knowledge. Moreover, she described this discourse activity as an ongoing process of reflection and action characterised by asking questions, seeking feedback, experimenting, reflecting on results and discussing errors or unexpected outcomes of actions.^2^, ^3^ Elaborating on this concept, Van den Bossche et al^4^ defined team learning as the discourse and its characteristics that ultimately lead to collectively developed cognition: shared cognitions or shared mental models, including shared representations of tasks, working relationships and situations. In their integrative and interdisciplinary review, Decuyper et al^5^ argued that team learning is about knowledge acquisition, participation in the team and the creation of new knowledge. As such, they defined team learning to be ‘*a compilation of team‐level processes that circularly generate change or improvement for teams, team members, organizations, etc*’.^5^ Based on these descriptions, we too view team learning as a team‐level process.^2^, ^3^, ^4^, ^5^ More specifically, when we say team learning, we refer to patterns in the discourse activity of the team: the behaviours that team members engage in and that create better outcomes not only for individual team members (eg, competence development), but also for their team (eg, shared cognition) and their organisation (eg, knowledge or products).

As social processes are, in essence, at the heart of team learning, the concept has often been referred to as team learning behaviour. Team learning behaviour encompasses three basic behavioural dimensions that can be viewed as interactions focusing on sharing, co‐construction and constructive conflict.^5^ ‘Sharing’ describes the exchange of knowledge and experiences, perspectives and opinions. This behaviour has been found crucial to increase the effectiveness of teams.^4^ ‘Co‐construction’ takes place when team members ‘build on’ their mutual knowledge and experiences, by incorporating and accumulating their various individual representations. However, this happens only when they understand each other's representation of knowledge and ideas, and consequently accept these. Lastly, ‘constructive conflicts’ are created when, through discussion and negotiation, team members manage to integrate knowledge and ideas, ultimately leading to consensus or a new understanding shared by the team.^4^ In the example above, this would mean that the multidisciplinary team reaches an agreement on the current disease status and suitable treatment option(s) by means of negotiation.

If organisations apply this knowledge about team learning, their ‘units of interest’ will shift from individuals towards teams that work on knowledge products, research, care and strategies. This begs the question: If team learning is the proven prescription for team success, can we diagnose and treat unsuccessful teams? To answer this question, we must first realise that, for team learning to occur, individuals must work together in a real *team*: ‘*A collection of individuals who are interdependent in their tasks, who share responsibility for outcomes, who see themselves and who are seen by others as an intact social entity embedded in one or more larger social systems.*’^6^ However, not all teams consist of members that function interdependently and feel responsible for the team or its task as a whole. Consequently, simply giving a group of people a task does not automatically mean they will work together as a team. Unsuccessful teams lack a collective discourse and, therefore, ‘team learning’ remains a bridge too far.^7^

The potential of teams lies in achieving better understanding of problems and creating innovative ideas for improvements or solutions together. Paradoxically, teams that learn can also be ineffective by inadvertently adapting to unexpected conditions or situations that do not enhance their work.^5^ Moreover, disagreements can sometimes lead to conflicts, consequently causing harm to teams and detract from team performance. Conflicts that create personal, emotional or even hostile disagreements appear especially problematic.^8^ Although (inter)personal conflicts can jeopardise team learning, constructive conflicts have appeared vital for the development of a shared cognition, leading to better team performance.^4^ Shared mental models will only be constructed if diverse information and viewpoints are clarified, processed, understood, negotiated, and lead to convergence of meaning and a shared view on the topic.^4^ In order to transition from conflict to constructive conflict, it is recommended to encourage members’ interdependence, democratise power hierarchy, and create safe environments in interpersonal contexts.^2^, ^8^, ^9^ To conclude, proficiency in team learning requires willingness to think differently and incorporate other people's perspectives, while not completely shying away from conflict.^2^, ^8^, ^9^

Finally, it is important to take into account that team learning processes are not linked in a linear fashion. Instead, team learning is dynamic, as it depends on various variables such as leadership, boundary crossing and psychological safety, that are emergent and subject to changes.^5^ This also means that team learning captures different conditions that are needed for effective team performance. Therefore, to diagnose and treat unsuccessful teams, we must determine the presence or absence of team learning by first asking ourselves: Does the team display behavioural elements of sharing, co‐construction and ‐ especially ‐ constructive conflicts, and if not, how can these elements be improved?

In much of our daily practice, teams must engage in activities to serve a shared purpose. However, it becomes clear that teams should be handled with care. The concept of team learning provides tools to better understand team interactions, and allows us to recognise difficulties and improve team functioning. More importantly, team learning can result in outcomes that single individuals or people working in parallel would never be able to achieve, such as improved learning, teaching, research and medical care.
